# The role of a *FADS1* polymorphism in the association of fatty acid blood levels, BMI and blood pressure in young children—Analyses based on path models

**DOI:** 10.1371/journal.pone.0181485

**Published:** 2017-07-21

**Authors:** Maike Wolters, Carmen Dering, Alfonso Siani, Paola Russo, Jaakko Kaprio, Patrizia Risé, Luis A. Moreno, Stefaan De Henauw, Kirsten Mehlig, Toomas Veidebaum, Denés Molnár, Michael Tornaritis, Licia Iacoviello, Yannis Pitsiladis, Claudio Galli, Ronja Foraita, Claudia Börnhorst, on behalf of the IDEFICS and I. Family consortia

**Affiliations:** 1 Leibniz Institute for Prevention Research and Epidemiology—BIPS, Bremen, Germany; 2 Epidemiology and Population Genetics, Institute of Food Sciences, National Research Council, Avellino, Italy; 3 Institute for Molecular Medicine FIMM, University of Helsinki, Helsinki, Finland; 4 DiSFeB, Department of Pharmacological and Biomolecular Sciences, University of Milan, Milan, Italy; 5 GENUD (Growth, Exercise, Nutrition and Development) Research Group, Instituto Agroalimentario de Aragón (IA2), Instituto de Investigación Sanitaria Aragón (IIS Aragón), Centro de Investigación Biomédica en Red Fisiopatología de la Obesidad y Nutrición (CIBERObn), University of Zaragoza, Zaragoza, Spain; 6 Department of Public Health, Faculty of Medicine and Health Sciences, Ghent University, Ghent, Belgium; 7 Section for Epidemiology and Social Medicine, Institute of Medicine, Sahlgrenska Academy, University of Gothenburg, Gothenburg, Sweden; 8 National Institute for Health Development, Tallinn, Estonia; 9 National Institute of Health Promotion, University of Pécs, Pécs, Hungary; 10 Research and Education Institute of Child Health, Strovolos, Cyprus; 11 Department of Epidemiology and Prevention, IRCCS Istituto Neurologico Mediterraneo Neuromed, Pozzilli (IS), Italy; 12 University of Brighton, Eastbourne, United Kingdom; Wake Forest School of Medicine, UNITED STATES

## Abstract

**Background:**

The recent obesity epidemic in children also showed an increase in the prevalence of hypertension. As blood pressure (BP) is associated with (long-chain) polyunsaturated fatty acids (LC PUFA), genetic variation in desaturase enzymes being involved in the synthesis of LC PUFA may be associated with BP. This study aimed to investigate the direct effects (independent of mediating variables) and indirect effects (mediated through intermediate variables) of a common variant in the *FADS1* gene, rs174546, known to affect delta-5 desaturase (D5D) activity on PUFA level, body mass index (BMI) and BP.

**Methods:**

A subsample of the IDEFICS (Identification and prevention of dietary- and lifestyle-induced health effects in children and infants) baseline survey including 520 children aged 2 to <10 years from six European countries was included. The association between rs174546 (T<C) and BP z-score as well as the mediating effects of selected key PUFA levels (dihomo-gamma-linolenic acid, DGLA; arachidonic acid, ARA; eicosapentaenoic acid, EPA) or estimated D5D activity (D5D index) and BMI z-score were investigated through path model analyses, adjusting for sex, age, educational level of parents, family history of hypertension, lifestyle factors and blood levels of saturated and monounsaturated fatty acids, triglycerides and low density lipoprotein cholesterol. Whole blood fatty acids were measured by a validated gas chromatographic method and recorded as percentage of weight of all fatty acids detected.

**Results:**

Minor allele carriers of the SNP rs174546 had significantly higher DGLA and lower ARA and EPA levels as well as a lower D5D index. Via ARA and BMI z-score, the polymorphism had an indirect lowering effect on systolic BP z-score for each additional T allele (standardized effect estimate -0.057, p = 0.007). For DGLA, EPA and D5D index, the indirect effects of rs174546 on systolic BP were also negative but did not reach significance. DGLA and EPA had an increasing indirect effect on systolic BP via BMI. Results for diastolic BP were in general similar but effect estimates were lower compared to systolic BP.

**Conclusion:**

Genetic variation in *FADS1* influences BP via ARA and BMI indicating a favorable effect of the minor allele in SNP rs174546. Thus, polymorphisms with an impact on the D5D activity may play a role for the BP level mediated through PUFA and BMI. Therefore, health effects of dietary n-6 and n-3 PUFA may vary depending on genetic *FADS1* variants.

## Introduction

Blood pressure levels and the prevalence of hypertension are increasing in children and adolescents, particularly because of the epidemic of overweight and obesity already observed in young age groups [1, 2]. Blood and tissue levels of polyunsaturated fatty acids (PUFA), in particular long-chain (LC) PUFA have been shown to be associated with blood pressure (BP). Furthermore, circulating levels of n-3 LC PUFA were associated with a lower prevalence of hypertension [3, 4]. LC PUFA levels depend on dietary intake, bioavailability and PUFA metabolism. In the biosynthesis of LC PUFA from precursor PUFA, elongase and desaturase enzymes are crucial. *FADS1*, a member of the fatty acid (FA) desaturase gene cluster, codes for the enzyme delta-5 desaturase (D5D) which is involved in one step of the conversion of linoleic acid (LA, 18:2n-6) and alpha-linoleneic acid (ALA, 18:3n-3) to arachidonic acid (ARA, 20:4n-6) and eicosapentaenoic acid (EPA, 20:5n-3), respectively. Genetic variation in desaturases has shown to affect PUFA and LC PUFA tissue status and studies indicate that polymorphisms in this gene cluster also influence health outcomes like the lipoprotein profile and the risk of cardiovascular diseases [5]. Accordingly, estimated D5D activity (D5D index), calculated from the FA product/precursor ratio (20:4n-6/20:3n-6) was observed to be inversely associated with BMI [6–8] and BP [6, 9]. Thus, high D5D activity may be protective against adiposity and hypertension both of which are increasingly prevalent already in children and adolescents [10, 11] and tend to track into adulthood [12–14].

Additionally, D5D activity strongly influences the PUFA status [15], particularly blood levels of EPA, docosahexaenoic acid (DHA, 22:6n-3) and ARA which have been linked to BP [3, 16–18]. N-6 and n-3 LC PUFA can influence BP in several ways as they are precursors of prostaglandins, thromboxanes and oxidation products with BP modulating effects. While high ARA status has been shown to increase BP [4, 18, 19], n-3 LC PUFA seem to have BP lowering effects as indicated by several studies [3, 4, 20]. However, results of observational studies regarding n-3 LC PUFA and BP in children are inconsistent [18, 21, 22].

As polymorphisms in the *FADS1* gene are associated with circulating PUFA levels [23–27], the relation between LC PUFA and BP may be influenced by genetic variation. The single nucleotide polymorphism (SNP) rs174546 [24] was shown to have strong effects on serum PUFA levels. Carriers of the rs174546 minor (T) allele showed a reduced activity of D5D resulting in increased levels of the precursor n-6 PUFA LA and dihomo-gamma-linolenic acid (DGLA, 20:3n-6) as well as the n-3 precursor PUFA ALA while the n-6 and n-3 D5D products ARA and EPA were found to be decreased [24]. Several studies suggested that rs174546 plays an important functional role in the *FADS1* gene cluster [24, 27, 28]. Results of the HELENA study investigating minor alleles of nine SNPs in the *FADS* gene cluster in a large group of adolescents suggest that the rs174546 SNP is in linkage disequilibrium with a functional SNP within *FADS1* as all haplotypes carrying the minor allele were associated with lower D5D activity [24]. Also other studies indicated rs174546 to have a functional role in the *FADS1* gene variants or to be at least an appropriate proxy for a functional but yet unknown *FADS1* variant [27, 28]. Thus, we hypothesize that genetic variation in the *FADS1* rs174546 influences BP via whole blood PUFA levels and BMI as mediators, and we investigate the direct and indirect effects on BP applying path analysis.

## Methods

### Study population

The IDEFICS (Identification and prevention of dietary- and lifestyle-induced health effects in children and infants) baseline survey (T0) included a population-based sample of 16,228 children aged 2 to 9.9 years from eight European countries (Belgium, Cyprus, Estonia, Germany, Hungary, Italy, Spain, Sweden) who were examined in 2007/2008. Follow-up examinations were conducted after two (T1) and six (T3, I.Family study) years; the design of this cohort study has been described in detail elsewhere [29, 30]. However, in this study, only cross-sectional data from the IDEFICS baseline survey were included as only at T0 a large number of children with FA data was available. In the IDEFICS study risk factors of overweight and obesity were investigated in young children and anthropometric and clinical examinations were conducted at each survey wave. Additionally, health characteristics and lifestyle behaviours were collected and biosamples were taken. Parents gave written informed consent prior to study participation and children gave oral consent prior to the examinations. All participating centers obtained ethical approval by the regional committees: Tallinn Medical Research Ethics Committee, Tallinn, Estonia; Ethics Committee of the University of Bremen, Bremen, Germany; Egészségügyi Tudományos Tanács, Pécs, Hungary; Azienda Sanitaria Locale Avellino Comitato Etico, Avellino, Italy; Regionala Etikprövningsnämnden i Göteborg, Gothenburg, Sweden; Comité Ético de Investigación Clínica de Aragón, Zaragoza, Spain.

### Genotyping and quality control (QC) of SNP data

Genomic DNA was extracted from saliva samples. Genotyping was conducted using the UK Biobank Axiom® 96-Array from Affymetrix (Santa Clara, USA). Genotype calling was performed with respect to Affymetrix’s best practice guideline including analysis with SNPolisher assuming a dish QC > 0.82 and QC call rate > 0.97. 40 individuals were excluded for sample call rate ≤ 0.98, ambiguous sex assignments, heterozygosity or as population outliers assessed by principal component analysis [31, 32].

An additive effect was assumed, with 0, 1, or 2 encoding the number of minor allele T present at SNP rs174546. The analyses were limited to *FADS1* as no genetic data on *FADS2* was available.

### Fatty acids

In a subsample of 2600 IDEFICS participants with an oversampling of overweight and obese children, FA profiles were analyzed [29]. Blood samples were obtained by collecting a drop of fasting blood from a fingertip or by venipuncture and were applied directly to an antioxidant-treated paper strip. FA in whole blood were separated and determined by a rapid gas-liquid chromatography method in a central laboratory as previously described [33, 34]. This method was validated by several laboratories [34–36] and whole blood was shown to be representative for the total FA pool as it includes FA from all lipid classes with phospholipids of plasma and red blood cells (RBC) being the main contributors of the FA amount and profile [35]. Several studies confirmed that whole blood is a suitable marker for n-3 FA status and for long-term essential FA intake [34, 37, 38].

In the present analysis we used FA values of the n-3 PUFA, EPA and the n-6 PUFA, DGLA and ARA. FA are expressed as weight percentage of all FA detected (% wt/wt). Additionally, we calculated the D5D index from the product-precursor ratio (20:4n-6/20:3n-6).

### Blood pressure

A standardized protocol was used to measure systolic (SBP) and diastolic (DBP) BP with an automated oscillometric device (Welch Allyn, Inc., 4200B-E2, Skaneateles Falls, NY, USA) after at least 5 minutes of rest in seated position at the right arm with a cuff of appropriate size for arm circumference [39]. Two recordings were taken, with a 2-min interval between each, plus a third measurement in case of a >5% difference between the first two readings. As outcome measure the mean value of the two measurements with the smallest difference was used. BP was set to missing if the first and second measurement deviated by >5% and no third measurement value was available. Age, sex and height specific reference values from the US Department of Health and Human Services, National Institutes of Health (NIH, 2005) [40] were applied to classify SBP, DBP and BP to normal BP, prehypertension and hypertension. Based on the NIH references age-, sex- and height-specific z-scores were calculated and used as outcome variables in the subsequent analysis.

### BMI

Body weight was measured in light underwear in fasting state on a calibrated scale accurate to 0.1 kg (Tanita BC 420 MA Tanita Europe GmbH, Sindelfingen, Germany). A calibrated stadiometer (Seca 225 stadiometer, Birmingham, UK) was used to measure height of the children to the nearest 0.1 cm. BMI was calculated as weight [kg] divided by height [m] squared and BMI z-scores were calculated based on Cole & Lobstein (2012) [41].

#### Covariates

Covariate data such as date of birth, dietary and lifestyle behaviors and socioeconomic information were assessed using questionnaires. In a personal interview the family history of diseases and the health conditions of the participating child were collected.

Sex of the child, age (continuous), country (six countries as categories: Estonia, Germany, Hungary, Italy, Spain, Sweden), maximum educational level of parents according to the International Standard Classification of Education (ISCED) [42] (three categories: low level, ISCED 0, 1 and 2; medium, ISCED 3 and 4; high, ISCED 5 and 6), sedentary behaviour (television and computer time (hours/week)), family history of hypertension (yes vs. no; as reported for biological parents/siblings) and birth weight (g) were considered as covariates. As indicator of dietary salt intake we used the weekly frequency of salty/snack food consumption which includes hamburger, hot dog, kebab, wrap, salty snacks such as savory pastries and fritters as well as cold cuts and ready to cook meat products. Further, the consumption frequency of fish (times/week) was considered in all analyses including EPA.

The weight percentages of our focus PUFA vary individually also depending on the proportions of the different lipid classes (phospholipids, triacylglycerols and cholesterylesters) in plasma, RBC, HDL and LDL. As total saturated (SFA) and total monounsaturated FA (MUFA) are the major FA with different distributions in these lipid classes [35], also weight percentages of SFA and MUFA of total FA were included as covariates. Their inclusion in the models aims to adjust for the different proportions of lipid classes. Additionally, we included blood levels of triglycerides (TG) and low-density lipoprotein cholesterol (LDL) as covariates. TG were directly measured with the ‘point-of-care analyser’ Cholestech LDX (Cholestech, Hayward, CA, USA) while LDL was calculated from Cholestech data as previously described [43].

### Analysis group

**Fig 1:**
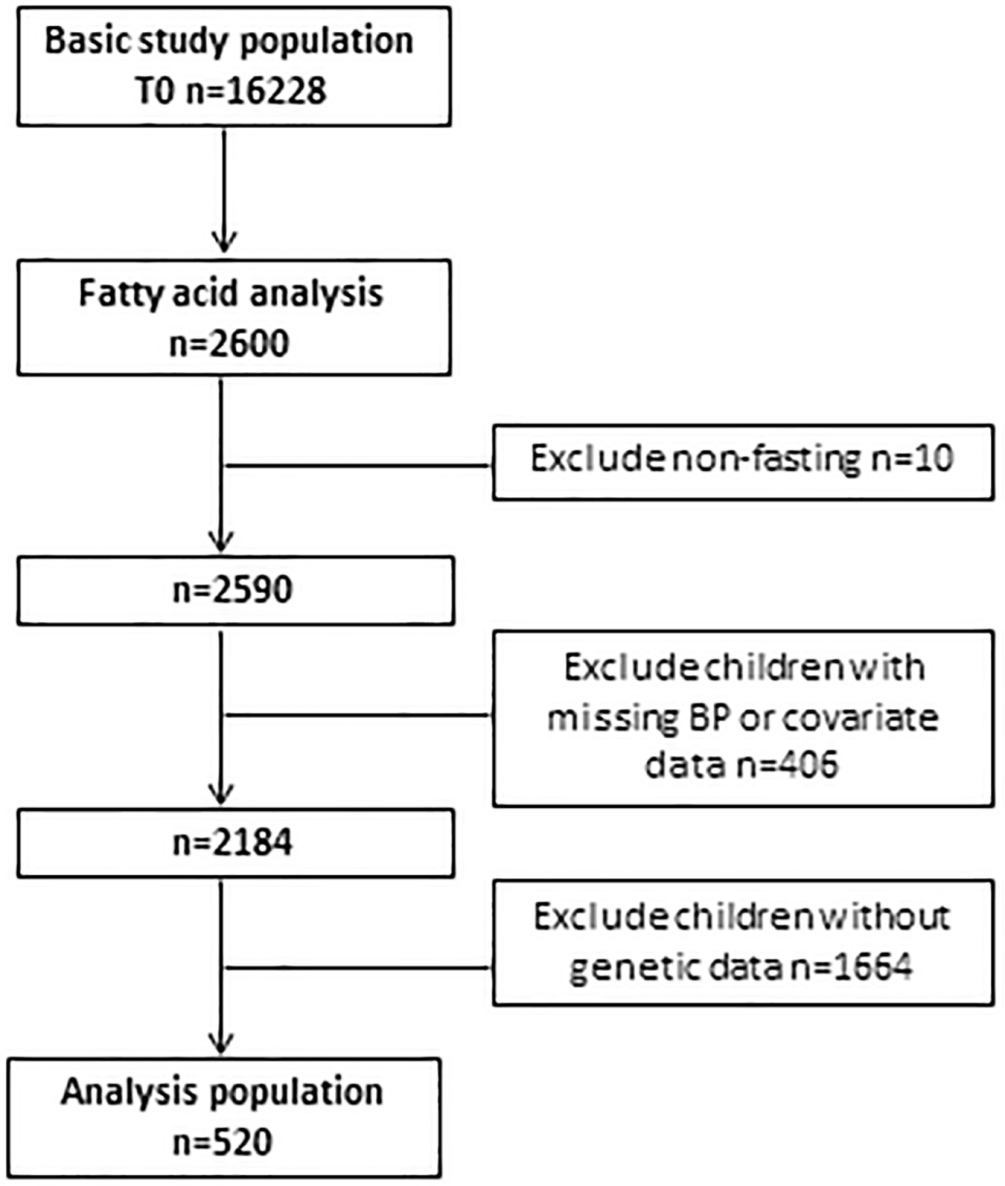
Flow chart of the inclusion and exclusion of IDEFICS participants.

Among the children with FA analyses (N = 2600), 10 children were excluded because of blood drawing in a non-fasting state and further 406 children had to be excluded due to missing BP or missing covariate data resulting in 2184 children. No genetic data was available for 1664 children, leaving 520 children in the final analysis sample (Fig 1).

### Statistical analyses

**Fig 2:**
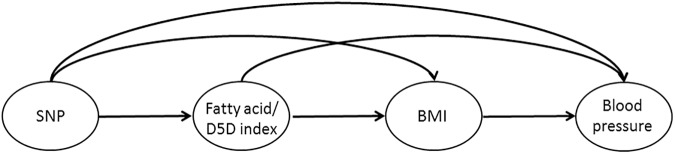
Theoretical models displaying the direct effects in proposed pathways through which BP (SBP or DBP, respectively) is affected by the SNP (rs174546), by fatty acids (DGLA, ARA, EPA, D5D index) as well as by BMI.

Path analysis was used to test a theory-driven model linking *FADS1* to blood pressure mediated by effects on FA and BMI. Path analysis has the advantage over linear regression that path coefficients are estimated via simultaneous equation estimation, thus adjusting standard errors for the number of equations computed [44]. These models further allow the estimation of total, direct and indirect effects. The structure of our theoretical model is displayed in Fig 2. The arrows in Fig 2 represent direct effects which are independent of the other variables in the path model. Additionally, there are indirect effects which influence a variable via an intermediate variable (not presented in Fig 2) [45]. This means that *FADS1* was assumed to affect BP directly but also indirectly through FA status (or D5D index) and BMI that lie on paths between *FADS1* and BP. The FA status (or D5D index) was assumed to affect BP directly as well as via BMI and the BMI was assumed to directly affect BP. Separate models were run for the two outcomes SBP and DBP as well as for the four exposures (1) DGLA, (2) ARA, (3) EPA and (4) D5D. Models 1a to 4a refer to the four models run for SBP, models 1b to 4b to the four models run for DBP, respectively. The covariates mentioned above were considered in the analyses (see S1 Table for a more detailed description of the covariates being related to the different endogenous (outcome) variables in the model). After running the *a priori* defined path model, the following model modifications were applied based on both theoretical considerations and model fit indices: covariances among residual terms, among country indicators and sex, among sex and age, sex and educational level of parents, educational level of parents and age, educational level of parents and SNP genotypes, SNP genotypes and age, SNP genotypes and media time as well as birth weight and media time were set to zero. Based on fit indices, also some changes in the covariates affecting the three endogenous variables BP, BMI and FA were applied if biologically plausible as summarized in S1 Table. For parameter estimation, we used the maximum-likelihood procedure with quasi-Newton optimization. A non-significant Chi-square test (P>0.05), Comparative Fit Index (CFI) ≥0.97, and Root Mean Square Error of Approximation (RMSEA) ≤0.03 indicated a very good model fit in all models estimated [46].

Mediation was assessed by computing estimates of direct, indirect, and total effects of the associations specified in the model. Direct effects represent associations between variables unmediated by any other variable in the model. Indirect effects represent mediated effects (or combined mediated effects for paths through multiple mediators). Total effects are the sum of the direct and indirect effects. For all models, standardized path coefficients and corresponding p-values are reported. Following the proposal by Cribbie (2007) [47] of how to account for multiple testing problems in pathway analysis, the adjustment method by Benjamini & Hochberg (1995) [48] was applied to control for the false discovery rate (FDR) at the 0.05 level of significance. Multiple comparison adjustment was applied for each outcome (SBP and DBP) and each research question resulting in different adjusted alpha levels which are indicated in the respective result table. All analyses were performed using SAS® statistical software version 9.3 (SAS Institute, Inc., Cary, NC). All path models were run using SAS Proc CALIS.

## Results

Table 1 gives a description of the total study group by blood pressure category. Children classified as hypertensive had a higher mean BMI z-score, a lower mean birth weight and a higher mean consumption frequency of salty/snack food compared with normotensive children. Table 2 shows the characteristics of the study population by rs174546 genotypes. The SNP was in Hardy-Weinberg Equilibrium (p = 0.395) and minor allele frequency for rs174546 was 29%. About 9% of children were homozygous and about 40% were heterozygous carriers of the minor allele.

**Table 1:** Baseline characteristics of the study population by blood pressure category^[1]^.

		All n = 520	Normal BP n = 425	Pre-hypertension n = 51	Hypertension n = 44
Sex, n (%)	Girls	260 (50.0)	214 (50.4)	21 (41.2)	25 (56.8)
	Boys	260 (50.0)	211 (49.6)	30 (58.8)	19 (43.2)
Age, years	Mean (SD)	6.45 (1.67)	6.52 (1.59)	5.69 (1.85)	6.67 (2.08)
	2-<6 years, n (%)	177 (34.0)	138 (32.5)	26 (51.0)	13 (29.5)
	6-<10 years, n (%)	343 (66.0)	287 (67.5)	25 (49.0)	31 (70.5)
BMI, Mean (SD)^[2]^	z-score	0.88 (1.27)	0.82 (1.26)	0.79 (1.33)	1.59 (1.10)
BMI category, n (%)^[2]^	Thin	29 (5.6)	26 (6.1)	2 (3.9)	1 (2.3)
	Normal weight	287 (55.2)	247 (58.1)	26 (51.0)	14 (31.8)
	Overweight	121 (23.3)	92 (21.6)	15 (29.4)	14 (31.8)
	Obese	83 (16.0)	60 (14.1)	8 (15.7)	15 (34.1)
*FADS1* rs174546,	CC	266 (51.2)	220 (51.8)	26 (51.0)	20 (45.5)
n (%)	CT	206 (39.6)	165 (38.8)	22 (43.1)	19 (43.2)
	TT	48 (9.2)	40 (9.4)	3 (5.9)	5 (11.4)
BP, Mean (SD)	SBP, z-score	0.41 (0.78)	0.19 (0.60)	0.94 (0.49)	1.89 (0.63)
	DBP, z-score	0.63 (0.59)	0.46 (0.46)	1.24 (0.38)	1.56 (0.60)
PUFA, % wt/wt, Mean (SD)	18:2n-6, LA	17.9 (2.01)	17.9 (1.97)	17.3 (1.83)	18.1 (2.47)
	20:3n-6, DGLA	1.22 (0.25)	1.22 (0.25)	1.23 (0.26)	1.24 (0.25)
	20:4n-6, ARA	7.53 (1.34)	7.50 (1.31)	7.70 (1.33)	7.70 (1.66)
	18:3n-3, ALA	0.20 (0.08)	0.20 (0.08)	0.19 (0.09)	0.20 (0.08)
	20:5n-3, EPA	0.27 (0.11)	0.27 (0.11)	0.25 (0.09)	0.31 (0.13)
	22:6n-3, DHA	1.20 (0.45)	1.18 (0.44)	1.18 (0.42)	1.35 (0.48)
D5D index, Mean (SD)	20:4n-6/20:3n-6	6.32 (1.29)	6.31 (1.30)	6.44 (1.18)	6.31 (1.35)
SFA, % wt/wt, Mean (SD)	sum	44.5 (1.93)	44.5 (1.95)	44.6 (1.96)	43.9 (1.66)
MUFA, % wt/wt, Mean (SD)	sum	25.1 (2.38)	25.0 (2.35)	25.3 (2.29)	24.9 (2.72)
Family history of hypertension, n (%)	no	413 (79.4)	341 (80.2)	39 (76.5)	33 (75.0)
	yes	107 (20.6)	84 (19.8)	12 (23.5)	11 (25.0)
Parental education, ISCED level, n (%)					
	Level 0, 1, 2	57 (11.0)	47 (11.1)	6 (11.8)	4 (9.1)
	Level 3, 4	297 (57.1)	244 (57.4)	28 (54.9)	25 (56.8)
	Level 5, 6	166 (31.9)	134 (31.5)	17 (33.3)	15 (34.1)
Birth weight, Mean (SD)	g	3352 (537)	3369 (531)	3303 (624)	3254 (488)
Consumption frequency of salty/snack food, Mean (SD)	times per week	5.34 (4.70)	5.24 (4.62)	5.55 (5.22)	6.11 (4.84)
Time spent with audiovisual media, Mean (SD)	hours per week	12.0 (7.70)	12.0 (7.81)	11.6 (6.76)	12.1 (7.77)

^[1]^National Institutes of Health 2005

^[2]^Cole & Lobstein 2012

ARA, arachidonic acid; ALA, alpha-linolenic acid; BMI, body mass index; BP, blood pressure; DBP, diastolic blood pressure; DHA, docosahexaenoic acid; DGLA, dihomo-gamma-linolenic acid; EPA, eicosapentaenoic acid; ISCED, International Standard Classification of Education; LA, linoleic acid; MUFA, monounsaturated fatty acids; PUFA, polyunsaturated fatty acids; SBP, systolic blood pressure; SD, standard deviation; SFA, saturated fatty acids; % wt/wt, weight percentage of all fatty acids detected.

**Table 2:** Baseline characteristics of the study population by *FADS1* genotype.

		*FADS1* rs174546		
		CC	CT	TT
All	n (%)	266 (51.2)	206 (39.6)	48 (9.2)
Sex, n (%)	Girls	118 (45.4)	115 (44.2)	27 (10.4)
	Boys	148 (56.9)	91 (35.0)	21 (8.1)
Age, years	Mean (SD)	6.45 (1.67)	6.52 (1.65)	6.13 (1.77)
	2-<6 years, n (%)	89 (50.3)	68 (38.4)	20 (11.3)
	6-<10 years, n (%)	177 (51.6)	138 (40.2)	28 (8.2)
BMI, Mean (SD)^[1]^	z-score	0.95 (1.29)	0.82 (1.24)	0.73 (1.26)
BP, Mean (SD)	SBP, z-score	0.35 (0.76)	0.47 (0.81)	0.44 (0.71)
	DBP, z-score	0.62 (0.59)	0.63 (0.58)	0.71 (0.60)
PUFA, % wt/wt, Mean (SD)	18:2n-6, LA	17.5 (1.86)	18.2 (2.13)	18.5 (1.89)
	20:3n-6, DGLA	1.16 (0.23)	1.25 (0.22)	1.43 (0.33)
	20:4n-6, ARA	7.81 (1.33)	7.34 (1.34)	6.88 (1.06)
	18:3n-3, ALA	0.19 (0.07)	0.21 (0.09)	0.21 (0.10)
	20:5n-3, EPA	0.27 (0.11)	0.26 (0.11)	0.25 (0.09)
	22:6n-3, DHA	1.22 (0.45)	1.18 (0.45)	1.13 (0.40)
D5D index, Mean (SD)	20:4n-6/20:3n-6	6.86 (1.18)	5.94 (1.08)	4.98 (1.11)

^[1]^Cole & Lobstein 2012

Pairwise correlations among the SNP, PUFA, BMI z-score, SBP z-score and DBP z-score are displayed in Table 3. Significant positive correlations were observed between BMI z-score and SBP z-score, DGLA and ARA. DGLA was positively correlated with the SNP. In contrast, the number of T alleles at SNP rs174546 correlated inversely with ARA and D5D index.

**Table 3:** Pearson correlation coefficients and p-values among SBP, DBP, BMI z-score, FA and D5D index and the SNP.

	SBP z-score	DBP z-score	BMI z-score	DGLA	ARA	EPA	D5D	rs174546
**SBP z-score**	1.00							
								
**DBP z-score**	**0.62**	1.00						
	< .0001							
**BMI z-score**	**0.28**	0.04	1.00					
	< .0001	0.360						
**DGLA**	0.04	-0.08	**0.23**	1.00				
	0.365	0.060	< .0001					
**ARA**	0.05	-0.05	**0.21**	**0.44**	1.00			
	0.294	0.284	< .0001	< .0001				
**EPA**	0.04	0.09	-0.02	-0.08	-0.04	1.00		
	0.387	0.040	0.693	0.060	0.384			
**D5D**	-0.02	0.04	-0.04	**-0.60**	**0.42**	0.05	1.00	
	0.738	0.356	0.410	< .0001	< .0001	0.266		
rs174546*	0.07	0.04	-0.04	**0.27**	**-0.22**	-0.09	**-0.48**	1.00
	0.120	0.420	0.328	< .0001	< .0001	0.041	< .0001	

*Spearman correlation

Bold figures indicate a false discovery rate (FDR) <0.05 for reported correlations. An FDR adjusted significance value corresponds to α_adj_ = 0.017.

Table 4 shows the estimated direct and indirect effects obtained from the different path models to disentangle the associations among BP z-score, BMI z-score, the FA and D5D index as well as the SNP rs174546. Models 1a to 4a present the results obtained for SBP with regard to DGLA (model 1a), ARA (model 1b), EPA (model 1c) and D5D (model 1d), and models 1b to 4b present the respective results for DBP. Standardized direct as well as indirect effects are displayed according to the defined path model. As expected, rs174546 showed significant direct effects on all FA levels and D5D index being positive for DGLA and negative for ARA, EPA and D5D index. The direct effects of the SNP on BMI were negative in all models but reached statistical significance only in the models for DGLA and for the D5D index. Neither the SNP nor the FA or D5D index showed a direct effect on SBP or DBP in any of the models. A direct effect on SBP and DBP was only observed for the BMI z-score. DGLA and EPA showed a positive direct effect on BMI.

**Table 4:** Standardized estimates of direct and indirect effects and corresponding p-values obtained from path analyses for the associations among systolic/diastolic blood pressure z-score, BMI z-score, the FA and D5D index and the SNP rs174546.

	DGLA (20:3n-6)		ARA (20:4n-6)		EPA (20:5n-3)		D5D index	
	Coefficient	p-value	Coefficient	p-value	Coefficient	p-value	Coefficient	p-value
**SBP**	Model 1a		Model 2a		Model 3a		Model 4a	
Direct effects on FA*								
rs174546	**0.353**	< .0001	**-0.237**	< .0001	**-0.092**	0.016	**-0.527**	< .0001
**Direct effects on BMI**								
FA*	**0.164**	0.001	0.076	0.243	**0.147**	0.001	-0.097	0.040
rs174546	**-0.134**	0.002	-0.058	0.167	-0.061	0.122	**-0.125**	0.007
**Direct effects on SBP**								
BMI	**0.288**	< .0001	**0.282**	< .0001	**0.284**	< .0001	**0.291**	< .0001
FA*	-0.002	0.968	0.149	0.040	0.027	0.576	0.035	0.482
rs174546	0.043	0.352	0.077	0.085	0.044	0.288	0.061	0.217
**Indirect effects**								
rs174546 —> FA* —> BMI	**0.058**	0.002	-0.018	0.247	-0.014	0.052	0.051	0.042
rs174546 —> FA* —> BMI—> SBP	-0.023	0.328	**-0.057**	0.007	-0.024	0.060	-0.040	0.170
FA* —> BMI—> SBP	**0.047**	0.004	0.021	0.251	**0.042**	0.004	-0.028	0.051
**DBP**	Model 1b		Model 2b		Model 3b		Model 4b	
Direct effects on FA*								
rs174546	**0.353**	< .0001	**-0.237**	< .0001	**-0.092**	0.016	**-0.527**	< .0001
**Direct effects on BMI**								
FA*	**0.164**	0.001	0.076	0.243	**0.147**	0.001	-0.097	0.040
rs174546	**-0.134**	0.002	-0.058	0.167	-0.061	0.122	**-0.125**	0.007
**Direct effects on DBP**								
BMI	**0.139**	0.002	**0.134**	0.003	**0.134**	0.003	**0.141**	0.002
FA*	-0.003	0.954	0.103	0.158	0.031	0.512	0.036	0.481
rs174546	-0.006	0.904	0.017	0.698	-0.004	0.922	0.012	0.804
**Indirect effects**								
rs174546 —> FA* —> BMI	**0.058**	0.002	-0.018	0.247	-0.014	0.052	0.051	0.042
rs174546 —> FA*—> BMI—> DBP	-0.012	0.587	-0.035	0.061	-0.013	0.091	-0.029	0.291
FA* —> BMI—> DBP	0.023	0.028	0.010	0.278	0.020	0.029	-0.014	0.087

*FA is a place holder for the fatty acids and D5D index being considered in the different models as given in the headline of the table, i.e. DGLA, ARA, EPA and D5D index, respectively.

Bold figures indicate a false discovery rate (FDR) <0.05 for reported effects on SBP and DBP, respectively. An FDR adjusted significance value corresponds to α_adj_ = 0.023 and α_adj_ = 0.018, respectively.

**Fig 3:**
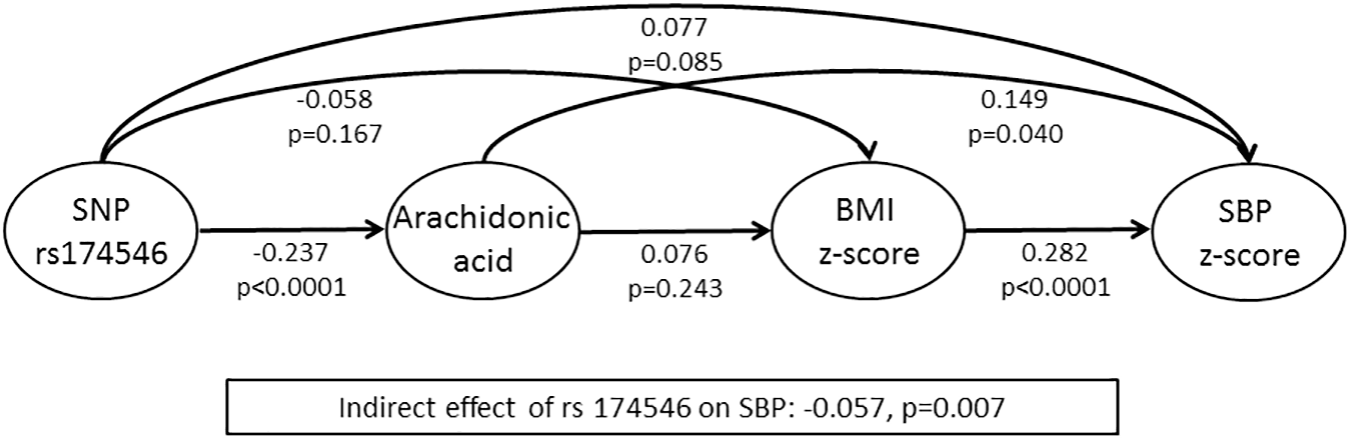
Path model for the associations between the SNP rs174546 and SBP z-score via ARA and BMI z-score: Standardized direct effects and p-values.

SNP rs174546 revealed a positive indirect effect on BMI via DGLA. A negative indirect effect of the SNP on SBP via ARA and BMI was observed. Fig 3 presents the estimated direct effects which are independent of mediating variables between the SNP rs174546, ARA, BMI and SBP. The largest effect observed was between BMI and SBP indicating that a higher BMI was associated with an increase in SBP. Further, there was a strong negative effect of the SNP on ARA indicating that in minor allele carriers the conversion from DGLA to ARA is reduced. As can be seen from Fig 3, the negative indirect effect between the SNP and SBP results from the negative direct effects of the SNP on ARA as well as on BMI overlaying the positive direct effects of ARA and the BMI on SBP.

DGLA and EPA had a positive indirect effect on SBP via BMI. The observed indirect effects on SBP point to the same directions for DBP.

The positive direct effect of EPA on BMI was unexpected and previous analyses indicated that the association of the EPA status with BP may depend on weight status [18]. Therefore, an additional analysis was conducted assessing the direct effects of EPA on BMI z-score and BP z-scores and the indirect effects of EPA on BP z-scores through BMI z-score separately for thin/normal weight versus overweight/obese children. This was done by use of respective interaction terms between weight status and EPA in the path model. The results are displayed in Table 5. EPA had a direct negative effect on BMI in thin/normal weight children whereas a positive effect was observed in overweight/obese children. The direct and indirect effects of EPA on BP point to the same directions, i.e. were negative in thin/normal weight children and positive in overweight/obese children.

**Table 5:** Standardized estimates of direct effects of EPA on BMI z-score and SBP z-score/DBP z-score and indirect effects of EPA on SBP z-score/DBP z-score via BMI z-score in thin/normal weight versus overweight/obese children obtained from path analyses.

	Thin/normal weight n = 316		Overweight/Obese n = 204	
**SBP**	Coefficient	p-value	Coefficient	p-value
**Direct effects on SBP**				
EPA	-0.042	0.568	0.155	0.039
**Direct effects on BMI**				
EPA	**-0.206**	< .0001	**0.535**	< .0001
**Indirect effects**				
EPA—> BMI—> SBP	-0.029	0.060	0.075	0.040
**DBP**				
**Direct effects on DBP**				
EPA	-0.023	0.756	0.143	0.058
**Direct effects on BMI**				
EPA	**-0.206**	< .0001	**0.535**	< .0001
**Indirect effects**				
EPA—> BMI—> DBP	-0.002	0.860	0.006	0.860

Bold figures indicate a false discovery rate (FDR) <0.05 for reported effects on SBP and DBP, respectively. An FDR adjusted significance value corresponds to α_adj_ = 0.025 for both models.

## Discussion

Our study in European children revealed that carrying the minor (T) variant of SNP rs174546, lying in the *FADS1* gene, has a favorable indirect effect on SBP via ARA in an analysis based on path models. However, no direct effect of the T allele on BP was observed. This indirect effect results from various countervailing direct effects via the blood level of ARA and the BMI z-score. The SNP was associated with lower ARA levels and this association may have overlaid the positive associations of ARA and the BMI with SBP in our model. To our knowledge this is the first study showing that the investigated *FADS1* SNP has a beneficial effect on SBP via its negative effects on the ARA level and on BMI z-score. This is in line with previous findings indicating that high ARA is associated with elevated BP in adults [4, 19] and, as observed in our recent analysis, also in children [18]. As expected, we observed strong negative effects of the SNP on ARA because in minor allele carriers the D5D-mediated conversion from DGLA to ARA is reduced. Further, the BMI z-score showed the expected positive association with BP.

While we observed a beneficial indirect effect of the SNP via ARA and the BMI on SBP, the direct association of the SNP with SBP indicated a positive direction which was not statistically significant (0.077, p = 0.085; total effect: 0.020, p = 0.641). Also the direct effects of ARA on BMI and the direct effects of ARA as well as BMI on SBP pointed to a positive direction although only the latter was statistically significant. The opposite direct effect of the SNP on SBP may result from factors not considered in the respective model which could have been influenced by the SNP, like the levels of other FA, e.g. decreased DHA [26] or increased insulin levels [6] in minor allele carriers, both of which can contribute to BP elevation [3, 16, 49].

For DGLA, EPA and D5D index, the indirect effects also pointed to a negative direction indicating a beneficial effect of the SNP though not being statistically significant. For DGLA, a beneficial indirect effect of the SNP on BP would be unlikely as more DGLA is available in minor allele carriers and DGLA had a positive indirect effect on BP in our study via BMI and was positively associated with BP in previous studies [17, 50, 51]. Additionally, high DGLA levels result in a lower D5D index which is the ratio of ARA and DGLA. The D5D index has shown to be inversely associated with BP in adults [6, 52]. Therefore, one may hypothesize that there is a positive association of rs174546 with BP via D5D index as minor allele carriers had a lower D5D index. However, because of the expected and observed strong negative direct effect of the SNP on the D5D index together with its negative direct effect on BMI, the resulting indirect effect of the SNP on SBP via D5D index and BMI pointed to a negative direction.

Additionally, our results suggest a positive indirect effect of the SNP on BMI via DGLA. The latter result is in line with a study in adolescents indicating a positive association of DGLA and BMI [53].

Contrary to our expectations, there was no significant indirect effect of the common *FADS1* SNP on BP via EPA and BMI. The SNP was assumed to be positively associated with BP via EPA as in children with minor alleles, EPA biosynthesis is reduced. High EPA levels were shown to be protective against hypertension in previous studies in adults [3, 4] although results in children are inconsistent [18, 21, 22]. In our model, EPA unexpectedly showed a direct positive effect on BMI and an indirect positive effect on SBP via BMI. However, a subgroup analysis revealed that the direct effect of EPA on BMI was only present in overweight/obese children whereas in thin/normal weight children the expected negative effect was observed. The indirect effect of EPA on BP also pointed to a positive effect in overweight/obese children but to a negative effect in thin/normal weight children. Thus, the unexpected effects of EPA are likely to result from effect modification by weight status. Also in a recent study, we found a beneficial effect of EPA on BP only in thin/normal weight children whereas an unfavorable association was observed in overweight/obese children [18]. We did not consider DHA in our analyses as the conversion efficiency from ALA and EPA to DHA is very low [54, 55] and we were particularly interested in the SNP’s influence on endogenous synthesis via *FADS1* and the effects on BP in this analysis. Additionally, in previous analyses no statistically significant effect of DHA on BP was observed neither in the total sample nor in an analysis stratified by weight status [18].

As reported in previous studies [23–27], our results confirm for the n-6 PUFA that the SNP directly affects the FA levels indicating that minor allele carriers had higher levels of precursor (DGLA) and lower levels of product FA (ARA) whereas for n-3 PUFA this effect is less obvious. This was also reflected by a negative effect on the D5D index in our study.

The rs174546 SNP has also shown to influence lipoprotein profiles as T allele carriers have lower cholesterol levels [56, 57]. Additionally, LDL and TG have been shown to influence the association between BMI and BP [58] and were hence included as covariates in the models. *FADS1* gene variants and cardiovascular diseases (CVD) or stroke for which hypertension is a major risk factor suggested a role of the rs174546 SNP. In a Chinese case-control study, the rs174546 T allele was found to be associated with an increased risk of coronary artery disease and ischemic stroke while the CC/CT genotypes were associated with a decreased risk [28]. In a large Danish study including more than 24,032 participants with 2648 CVD cases during 14 years of follow-up, no effect of rs174546 on CVD risk in the total study group was observed. However, among participants homozygous for the minor T allele of rs174546, subjects with a higher dietary intake of the n-3 FA ALA and a higher ratio of ALA to the n-6 FA LA tended to be at lower risk for CVD and ischemic stroke [59]. This may indicate a protective effect of the resulting higher EPA/ARA ratio. In our study the SNP had a negative effect on SBP via lower ARA. The observed trend for a negative effect of the SNP on SBP via EPA in the total study group depended on the weight status and indicated a protective role of EPA only in thin/normal weight children. The effects of variants of a common *FADS1* SNP on BP as shown in our study are one element that has to be considered in the complex association of n-6 and n-3 LC PUFA with cardiovascular risk factors.

### Limitations and strengths

Intrinsic variability of BP could not be considered in our study because repeated BP measurements could be taken only on a single occasion on the examination day. This may have led to an overestimation of BP values [40, 60]. As we had no reliable information on dietary sodium intake, we used the intake frequency of salty/snack foods as a proxy for sodium intake. These and other processed foods have shown to be the main source of salt [61]. As absolute quantitative data of FA were not available we included weight percentages of SFA and MUFA of total FA as covariates to adjust for the different proportions of lipid classes.

As all data were measured cross-sectionally in the present study, thus causality cannot be determined.

Important strengths of our study are the pan-European analysis sample of 520 children as well as the detailed phenotyping of the children. In all countries standardized protocols for data assessment, measurements and biosampling were applied and adherence to protocols was verified by central quality control. To investigate the association of the SNP and BP in children can be an advantage as the genetic basis may be stronger compared to adults because of the accumulation of behavioral and lifetime exposures in older indviduals [62, 63].

## Conclusions

Our results indicate that genetic variation in the *FADS1* gene, rs174546, influences BP via ARA and BMI. Thus, polymorphisms with an impact on the D5D activity may play a role for the BP level mediated through PUFA and BMI. Therefore, health effects of dietary n-6 and n-3 PUFA may vary depending on genetic *FADS1* variants and—considering the observed effect modification—on weight status. Different dietary intake recommendations for LC PUFA may be required according to genotype and weight status.

## Supporting information

S1 Table Exposures and covariates selected a priori for the three endogenous variables (BP, BMI z-score and FA and D5D index) as well as after model modification based on fit indices and theoretical considerations (final model) (DOCX)

## Abbreviations

ALA: alpha-linolenic acid
ARA: arachidonic acid
BMI: body mass index
BP: blood pressure
DBP: diastolic blood pressure
DHA: docosahexaenoic acid
DGLA: dihomo-gamma-linolenic acid
EPA: eicosapentaenoic acid
FA: fatty acids
IDEFICS study: Identification and prevention of dietary- and lifestyle-induced health effects in children and infants study
ISCED: International Standard Classification of Education
LA: linoleic acid
LC PUFA: long chain polyunsaturated fatty acids
MUFA: monounsaturated fatty acids
PUFA: polyunsaturated fatty acids
SBP: systolic blood pressure
SFA: saturated fatty acids
