# Supplementary material for: The role of a FADS1 polymorphism in the association of fatty acid blood levels, BMI and blood pressure in young children—Analyses based on path models
Source: PLoS One. 2017 Jul 21;12(7):e0181485. doi: 10.1371/journal.pone.0181485 (PMC5521833; doi:10.1371/journal.pone.0181485)
Supplement: S1 Table — (DOCX) [file pone.0181485.s001.docx]

**S1 Table: Exposures and covariates selected a priori for the three endogenous variables (BP, BMI z-score and FA and D5D index) as well as after model modification based on fit indices and theoretical considerations (final model)**

| **Endogenous variable** | **Exposures and covariates in a priori defined path model** | **Exposures and covariates in the final path model** |
| --- | --- | --- |
| SBP and DBP z-scores | SNP, FA, BMI z-score, age, sex, educational level of parents, country, consumption of salty/snack foods, family history of hypertension, average media time, birth weight, LDL, TG | SNP, FA, BMI z-score, age, sex, educational level of parents, country, consumption of salty/snack foods, family history of hypertension, average media time, birth weight, LDL, TG, SFA and MUFA levels |
| BMI z-score | SNP, FA, age, sex, educational level of parents, country, consumption of salty/snack foods, average media time, birth weight | SNP, FA, age, sex, educational level of parents, country, consumption of salty/snack foods, average media time, birth weight, SFA and MUFA levels, family history of hypertension |
| FA  (DGLA, ARA, EPA, D5D) | SNP, age, sex, educational level of parents, country, consumption of salty/snack foods (for DGLA, ARA and D5D only), average media time, birth weight, consumption of fish (for EPA only), SFA and MUFA levels, LDL, TG | SNP, age, sex, educational level of parents, country, consumption of salty/snack foods (for DGLA, ARA and D5D only), average media time, birth weight, consumption of fish (for EPA only), SFA and MUFA levels, LDL, TG |
